# A Bibliometric Analysis on Viral Central Nervous System Infection Research Productivity in Southeast Asia

**DOI:** 10.7759/cureus.35388

**Published:** 2023-02-23

**Authors:** Anna Anjelica R Sanchez, Roland Dominic G Jamora, Adrian I Espiritu

**Affiliations:** 1 Department of Internal Medicine, Section of Adult Neurology, Cardinal Santos Medical Center, San Juan, PHL; 2 Department of Clinical Neurosciences, Section of Neurology, University of the East Ramon Magsaysay Memorial Medical Center, Quezon, PHL; 3 Department of Neurosciences, Section of Neurology, Movement Disorders Services, St. Luke's Medical Center, Taguig, PHL; 4 Department of Neurosciences, College of Medicine, Philippine General Hospital, University of the Philippines Manila, Manila, PHL; 5 Department of Clinical Epidemiology, College of Medicine, University of the Philippines Manila, Manila, PHL; 6 Department of Neurology, St. Michael’s Hospital, University of Toronto, Toronto, CAN

**Keywords:** research performance, research productivity, southeast asia, bibliometrics, viral infections of the nervous system

## Abstract

Research productivity on viral infections of the nervous system in Southeast Asia (SEA) is unknown. We aimed to determine the research productivity of SEA in terms of bibliometric indices and PlumX metrics and their correlation with socioeconomic factors.

A comprehensive search of major electronic databases was done to identify studies on viral infections of the nervous system with at least one author from SEA. Socioeconomic factors and collaborations outside SEA were determined. Correlational analysis was done on bibliometric indices and socioeconomic factors.

A total of 542 articles were analyzed. The majority came from Thailand (n = 164, 30.2%). Most articles used a descriptive study design (n = 175, 32.2%). The most common topic was Japanese encephalitis (n = 170, 31.3%). The % gross domestic product allotted for research, number of neurologists, and number of collaborations outside SEA correlated with the bibliometric indices and PlumX metrics.

In conclusion, the number of research from SEA was low but the quality was comparable to the global benchmark. Improving resource allocation and collaboration between SEA nations and other countries may support this endeavor.

## Introduction and background

Viruses are important etiologic agents of nervous system infections not only because of their complex nature and neuropathogenesis but also because of their ability to cause epidemics and pandemics. These infections can present as meningitis, encephalitis, meningoencephalitis, myelitis, and acute flaccid paralysis. In Southeast Asia (SEA), viral infections of the nervous system are of special interest since this region has seen outbreaks and the emergence of viral encephalitides like Japanese encephalitis and Nipah encephalitis [1,2]. Re-emerging viral infections such as poliomyelitis and measles have also been reported in the region [3-5]. The current COVID-19 pandemic has also shown the virus to produce neurologic manifestations and sequelae [6,7].

This group of diseases causes significant morbidity and mortality in affected individuals and is associated with a high socioeconomic burden since they usually cause long-term disabilities [1,8]. The paucity of available treatment options, changing demographics, and a sub-optimal healthcare delivery system make the eradication of these diseases challenging.

Research performance has traditionally been measured in terms of research output, citations, assessment by expert panels, and in some, indicators of the reputation of researchers [9]. Other indicators which have been identified to measure the output and outcome of medical research include collaboration, industrial production, dissemination, and health service impact [10]. Altmetrics like Plum Analytics’ PlumX Metrics, which uses five broad categories: citations, usage, captures, mentions, and social media, are more immediate metrics compared to the traditional indicators. When used alongside each other, traditional and alternative metrics give a broader insight into the quality and impact of the research [11].

The research output of SEA on other neurological conditions such as headache, epilepsy, stroke, primary brain tumor, bacterial infections of the central nervous system, movement disorders, dementia, multiple sclerosis, motor neuron diseases, and neuromyelitis optica spectrum disorder have been studied and reported [12-20]. We aimed to determine the research performance of SEA countries on viral infections of the nervous system. This study evaluated the research performance of each SEA country in terms of research output, citations and dissemination, collaboration, and PlumX metrics. Associations between the research performance and socioeconomic factors including gross domestic product (GDP), % GDP allotted for research and developments as well as the number of neurologists were analyzed.

Methods

A systematic review was done to retrieve studies on viral infections of the nervous system. The Preferred Reporting Items for Systematic reviews and Meta-Analyses (PRISMA) guidelines were followed [21].

Criteria for Considering Studies for This Review

All published articles on viral infection of the nervous system with at least one author affiliated with any of the SEA countries using any study design such as clinical trials, laboratory studies, cohort studies, case-control studies, cross-sectional studies, descriptive studies, case series/reports, literature reviews, and systematic review or meta-analysis were included in the study. Conference papers, letters to the editor, book chapters, terminated studies, proceedings, commentaries, written in a non-English language, and abstract-only papers were excluded.

Search Methods for Identification of Studies and Selection Process of Studies

We performed a systematic search of articles in PubMed, Scopus, and the Western Pacific Regional Index Medicus (WPRIM) using the following search terms: ("viral infections of the nervous system" OR "viral meningitis" OR "viral encephalitis" OR "viral meningoencephalitis" OR "herpes simplex encephalitis" OR "dengue encephalitis" OR "Japanese encephalitis" OR "Nipah virus encephalitis" OR "Rabies" OR "Subacute sclerosing panencephalitis" OR "acute flaccid paralysis") AND ("Philippines" OR "Indonesia" OR "Malaysia" OR "Thailand" OR "Cambodia" OR "Brunei" OR "Singapore" OR "Laos" OR "Lao PDR" OR "Vietnam" OR "Myanmar" OR "Burma" OR "Timor-Leste" OR "East Timor" OR "Timor"). Articles from the earliest indexed record up to March 2021 were included.

Two reviewers (AARS and AIE) worked independently to screen the records and collect the data. The studies were screened based on title, author and author affiliation, and abstracts. Full texts of eligible studies were then downloaded and reviewed. Data was encoded into a Microsoft Excel file. Duplicate articles were removed. Studies that fulfilled the eligibility criteria were included in the quantitative and qualitative analysis.

Bibliometric Indices

From the identified studies, the following bibliometric indices were used: Number of publications, publications in journals with impact factor (IF), field-weighted citation impact (FWCI), and PlumX metrics.

The total number of publications was ascertained as the sum of all published articles per SEA country. Articles in collaboration with other SEA countries were assigned and counted to the countries involved in the study. Journal IF measures the yearly frequency of the average citation of an article in a journal. The higher the number of citations a journal receives via its published articles, the higher the IF [22]. The journal IF was determined based on the Journal Citation Reports by Clarivate Analytics [23].

The FWCI, which was obtained via the Scopus website, reflects the mean citation impact and compares it to the expected number of citations for studies of the same document type, year of publication, and subject area. A global baseline score of 1 means that the publication has been cited at the expected world average for similar publications. Scores greater than 1 indicate that the publications have been cited more than expected while those with scores less than 1 indicate that the study has been cited less than expected based on the world average [24,25].

PlumX designates metrics in five broad categories of indices namely: (a) Citations (traditional citation indexes, patent citations, and clinical citations), (b) Usage (clicks, downloads, and views), (c) Captures (read, exported, and saved), (d) Mentions (blog posts, comments, reviews, and news media), and (e) Social media (likes, tweets, and comments in various social media platforms). Data for PlumX metric indices were obtained from the Scopus website.

Socioeconomic Data and Collaborations

Data on each SEA country’s population, GDP per capita, and % GDP for research and development were acquired through the World Bank website. The number of neurologists per country was obtained based on recently published studies. The number of persons served by each neurologist was then computed by dividing the country’s total population by the number of neurologists. Collaborations with countries outside SEA were identified and counted based on author affiliation. For papers with authors from multiple SEA countries, collaborations were counted and ascribed to each SEA country involved in the study.

Data Analysis

The following information was extracted from the articles deemed eligible for the study: country and institutions where the author/s is/are affiliated, year of publication, the journal where the article was published, journal IF, study design, type of viral infection, research domain studied, collaboration with other countries outside SEA, source of funding, as well as the previously described research performance indices.

Statistical analyses were done using Statistical Product and Service Solutions (SPSS) (IBM SPSS Statistics for Windows, Version 26, Armonk, NY). For categorical values, data were expressed as frequencies and proportions. Correlations between the characteristics of each country and bibliometric indices were determined using the Spearman correlation coefficient. A value of p < 0.1 was considered statistically significant.

The authors confirm that this bibliometric study had been prepared in accordance with Committee on Publication Ethics (COPE) roles and regulations. Given the nature of a bibliometric study, the Institutional Review Board (IRB) review was not required.

Results

Study Selection

**Figure 1:**
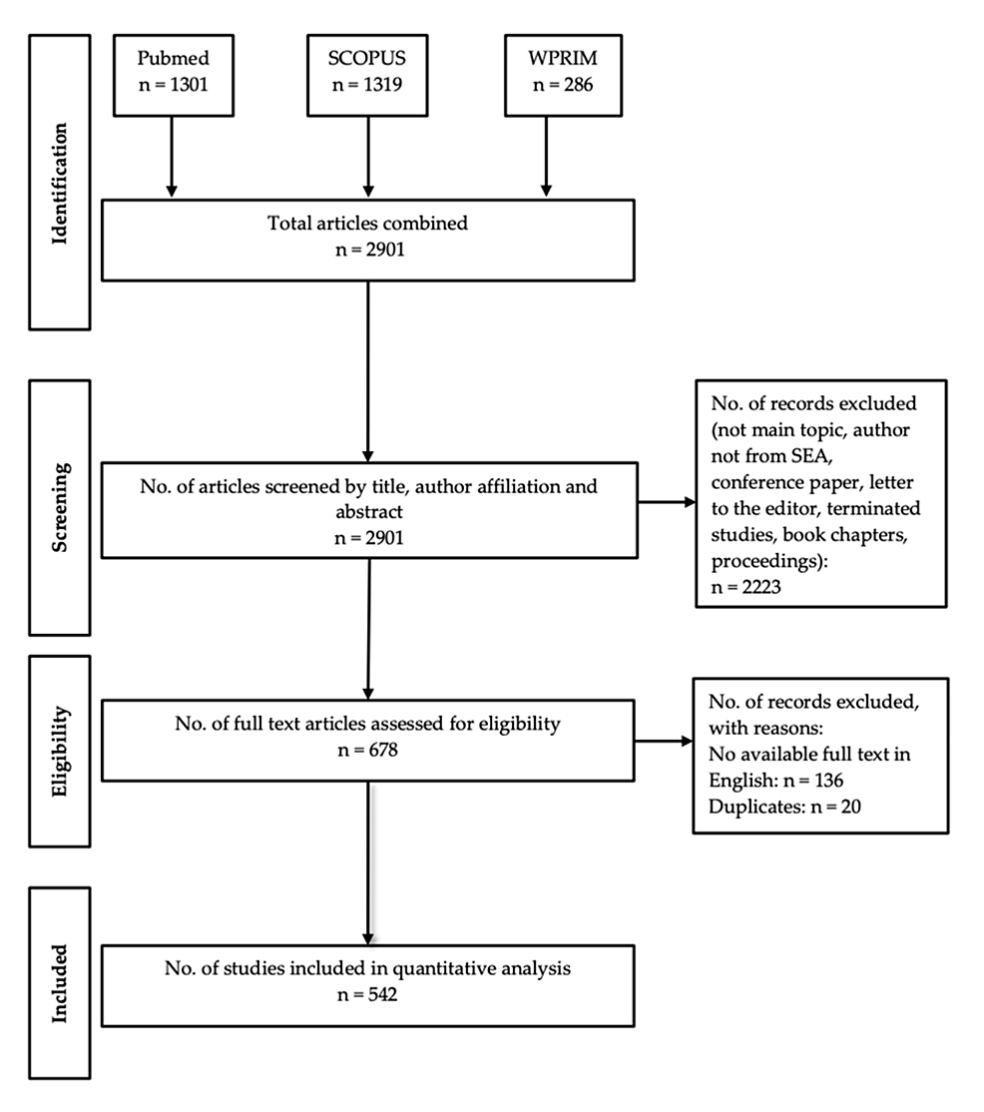
PRISMA flow diagram of study selection. SEA: Southeast Asia

A total of 2907 articles were identified from three electronic databases (Pubmed = 1301, Scopus = 1319, WPRIM = 286) until March 31, 2021. After screening based on title, abstract, and author affiliation, a total of 678 articles were identified and analyzed. A total of 136 articles that did not have an available full text in English and 20 duplicates were removed. Thus, 542 articles were included in the final analysis (Figure 1).

Number of Publications per SEA Country

Out of the SEA countries, Thailand (n = 164, 30.2%) has the most number of publications followed by Malaysia (n = 90, 16.6%), and the Philippines (n = 70, 12.9%). Myanmar (n = 7, 1.3%), Timor Leste (n = 1, 0.2%), and Brunei (n = 0, 0%) had the least number of publications.

Singapore (1.80), Malaysia (1.56), and the Philippines (1.22) have the highest average field-weighted index among the SEA countries. While Myanmar (0.15), Laos (0.67), and Indonesia (0.81) have the lowest average field-weighted index.

Characteristics of Published Journal Articles

The most common study design was descriptive (n = 175, 32.2%), followed by prospective cohort studies (n = 95, 17.2%), and laboratory studies (n = 66, 12.2%).

**Figure 2:**
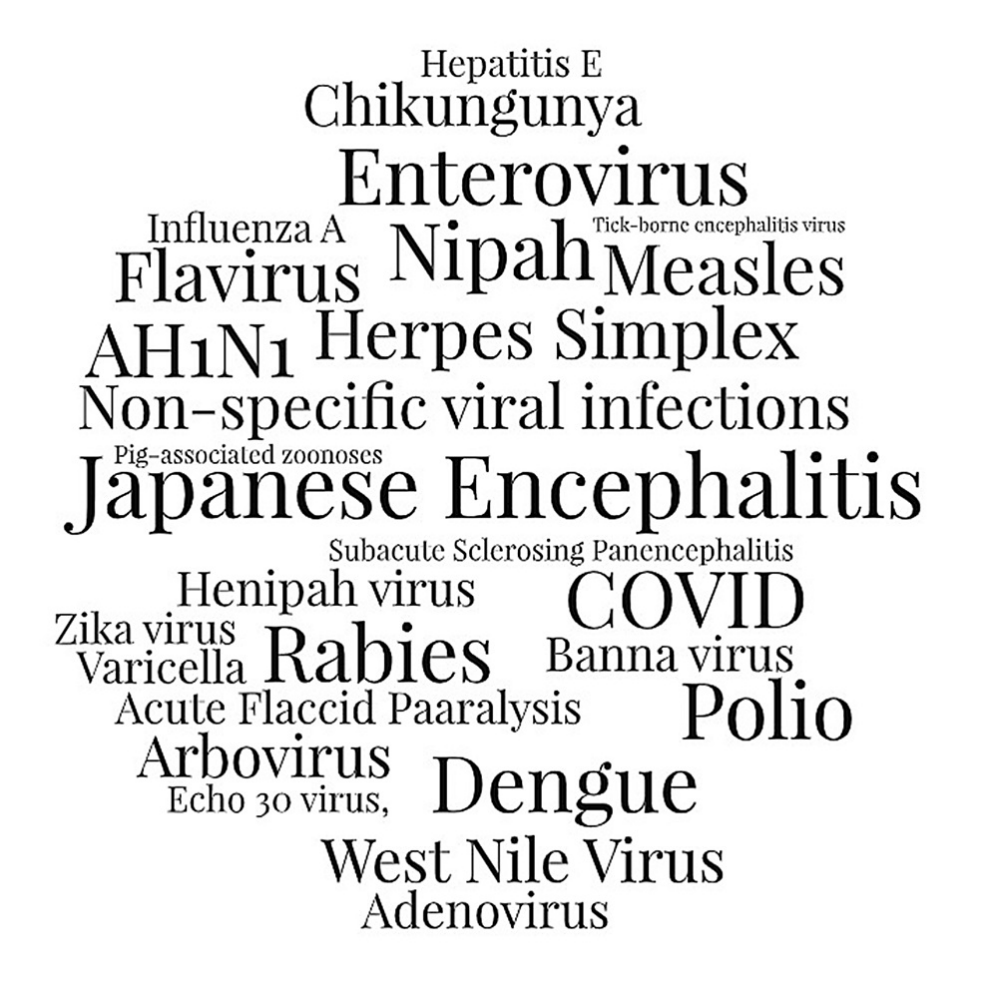
Word cloud of research topics.

Japanese encephalitis was the most common topic of this research (n = 170, 34.4%). This was followed by rabies (n = 168, 34%), and non-specific viral infections (n = 38, 7.7%). The least commonly studied topics were chikungunya, henipah virus, arbovirus, measles, AH1N1, adenovirus, Banna virus, echo 30 virus, hepatitis E, influenza A, pig-associated zoonoses, tick-borne encephalitis virus, varicella virus, flavivirus, and zika virus. A word cloud on the topics of these researches is seen in Figure 2.

The most studied research domains in SEA were pathophysiology (n = 126, 23.3%), prevention (n = 121, 22.3%), and epidemiology (n = 112, 20.7%). The least common were economics (n = 8, 1.5%); knowledge, attitude, and practices ( n = 8, 1.5%); and other types of studies which include timelines and future perspectives (n = 7, 1.3%).

Journals and Impact Factor

Out of 542 articles, 466 (86%) of studies from SEA were published in journals with IF. It is noteworthy that at least 80% of researches coming from Cambodia (n = 32/33, 97%), Thailand (n = 155/164, 94.5%), Laos (n = ­22/24, 91.7%), Vietnam (n = 61/67, 91.0%), Malaysia (n = 75/90, 83.3%), and Singapore (n = 36/43, 83.7%) were published in these journals. The top three journals with the most publications from SEA were PLOS Neglected Tropical Diseases (n = 39, 8.4%), Vaccine (n = 39, 8.4%), and the American Journal of Tropical Medicine and Hygiene (n = 28, 6.0%).

Thirteen articles (2.4%) were published in journals with an IF of more than 20. These journals were the New England Journal of Medicine (IF: 91.245, n = 2), Lancet (IF: 79.321, n = 6), Nature (IF: 49.962, n = 1), Nature Reviews Neurology (IF: 42.937, n = 1), BMJ (IF: 39.890, n = 2), and The Lancet Infectious Diseases (IF: 25.071, n = 1).

Altmetrics (PlumX Metrics)

Thailand (n = 4310) followed by Malaysia (n = 3250), and Vietnam (n = 1801) have the most number of citations. For usage, Vietnam (n = 129,112), the Philippines (n = 114,993), and Thailand (n = 52,855) scored the highest. For captures, Thailand (n = 6348), Malaysia (n = 4310), and Vietnam (n = 3281) have the highest numbers. Malaysia (n = 49), Vietnam (n = 22), and Laos (n = 19) have the most mentions. Thailand (n = 3130), the Philippines (n = 2399), and Indonesia (n = 692) have the most numbers when it comes to social media.

Socioeconomic Factors and Correlation with Bibliometric Indices

In SEA, the most populated countries (in thousands) are Indonesia (273,523.62), the Philippines (109,581.09), and Vietnam (97,338.58); while Singapore (5,685.81), Timor Leste (1,318.44), and Brunei (437.48) are the least populated [26]. Indonesia (1150), Vietnam (800), and Thailand (645) have the most number of neurologists in the country. However, in terms of population (in thousands) served per neurologist, Singapore (56.86), Thailand (108.22), and Vietnam (121.67) have the lowest population per neurologist ratio [15,26].

The countries in SEA with the highest GDP per capita (in USD) are Singapore (65,640.7), Brunei (27,466.3), and Malaysia (11,414.2). The percentage of GDP allotted for research and development is highest in Singapore (2.19%), Malaysia (1.26%), and Thailand (0.48%) and lowest in Laos, Brunei, and Cambodia [26].

GDP did not have a significant association with any bibliometric indices. However, a positive correlation was found between the % GDP allotted for research and development and the number of publications (r = 0.665, p = 0.036), the number of publications in journals with IF (r = 0.650, p = 0.042), and field weighted index (r = 0.748, p = 0.013). A strong positive correlation was found between the number of neurologists and the number of publications (r = -0.788, p = 0.004), and the number of publications in journals with IF (r = 0.827 p = 0.002) (Table 1).

**Table 1:** Correlation analysis of socioeconomic factors and research performance. *Strong correlation **Very strong correlation GDP: gross domestic product; IF: impact factor

Socio-economic factors	Research performance	Spearman’s Rho Coefficient	p value
GDP per capita	Number of publications	0.374	0.258
	Number of publications in journals with IF	0.345	0.298
	Field Weighted Index	0.418	0.201
Research and development	Number of publications	0.665	0.036*
	Number of publications in journals with IF	0.650	0.042*
	Field Weighted Index	0.748	0.013*
Number of neurologists	Number of publications	0.788	0.004**
	Number of publications in journals with IF	0.827	0.002*
	Field Weighted Index	0.382	0.247
Population per neurologist	Number of publications	0.486	0.154
	Number of publications in journals with IF	0.467	0.174
	Field Weighted Index	0.442	0.200

The percentage of GDP allotted to research and development was seen to have a positive correlation with the number of citations (r = 0.723, p = 0.018) while the number of neurologists has a positive correlation with usage (r = 0.709, p = 0.015). The number of neurologists in the country was also seen to be strongly correlated to citations (r = 0.745, p = 0.008), captures (r = 0.809, p = 0.003), and social media (r = 0.806, p = 0.003) (Table 2).

**Table 2:** Correlation analysis of socioeconomic factors and altmetrics. * Strong Correlation ** Very Strong Correlation GDP: gross domestic product

Socioeconomic factors	Altmetrics	Spearman’s Rho Coefficient	p value
GDP per capita	Citation	0.391	0.235
	Usage	0.036	0.915
	Captures	0.273	0.417
	Mentions	0.202	0.552
	Social Media	0.155	0.649
% GDP allotted to research and development	Citation	0.723	0.018*
	Usage	0.067	0.854
	Captures	0.565	0.089
	Mentions	0.326	0.358
	Social Media	0.158	0.663
Number of neurologists	Citation	0.745	0.008**
	Usage	0.709	0.015*
	Captures	0.809	0.003**
	Mentions	0.459	0.156
	Social Media	0.806	0.003**
Population per neurologist	Citation	0.527	0.117
	Usage	0.236	0.511
	Captures	0.358	0.310
	Mentions	0.049	0.894
	Social Media	0.297	0.405

Collaborations and Correlation with Bibliometric Indices

Most collaborations were with European countries (n = 202), such as the United Kingdom (n = 90) and France (n = 71). This was followed by other Asian countries outside SEA (n = 125), such as Japan (n = 60), China (n = 13), and South Korea (n = 12) (Table 3).

**Table 3:** Collaborations with countries outside Southeast Asia.* * Counted and ascribed to each Southeast Asian country involved in the study.

Country	North America	Europe	Oceania	Asia	South America	Africa
Thailand	n = 42	n = 40	n = 4	n = 34	n = 0	n = 4
	USA: 37 Canada: 5	United Kingdom: 19 France: 18 Germany: 3	Australia: 3 New Zealand: 1	Japan: 18 China: 4 South Korea: 3 India: 2 Sri Lanka: 2 Taiwan: 2 Bhutan: 1 Hong Kong: 1		Gabon: 3 Kenya: 1
Malaysia	n = 12	n = 17	n = 3	n = 22	n = 0	n = 0
	USA: 11 Canada: 1	United Kingdom: 14 France: 1 Germany: 1 Turkey: 1	Australia: 1 New Zealand: 1	Japan: 5 India: 3 South Korea: 2 Taiwan: 2 Japan: 2 China: 1 Bangladesh: 1 Hong Kong: 1		
Philippines	n = 18	n = 19	n = 4	n = 23	n = 0	n = 1
	USA: 18	France: 10 Germany: 2 United Kingdom: 2 Switzerland: 1 Turkey: 1 Ireland: 1 Austria: 1 Belgium: 1	Australia: 3 New Zealand: 1	Japan: 16 India: 2 China: 2 Saudi Arabia: 1 Taiwan: 1 South Korea: 1		Cameroon: 1
Vietnam	n = 18	n = 37	n = 3	n = 20	n = 1	n = 0
	USA: 17 Canada: 1	United Kingdom: 21 France: 6 Netherlands: 6 Norway: 2 Switzerland: 1 Germany: 1	Australia: 3	Japan: 16 South Korea: 2 China: 1 Taiwan: 1	Brazil: 1	
Singapore	n = 11	n = 18	n = 4	n = 9	n = 0	n = 0
	USA:11	France: 11 United Kingdom: 3 Netherlands: 2 Belgium: 1 Switzerland: 1	Australia: 2 New Zealand: 2	China: 2 India: 2 Japan: 2 Taiwan: 1 South Korea: 1 Hong Kong: 1		
Indonesia	n = 10	n = 10	n = 4	n = 9	n = 0	n = 0
	USA: 9 Canada: 1	United Kingdom: 6 Netherlands: 4	Australia: 3 New Zealand: 1	South Korea: 3 Japan: 2 China: 2 Taiwan: 1 India: 1		
Cambodia	n = 10	n = 19	n = 5	n = 6	n = 0	n = 0
	USA: 8 Canada: 2	France: 12 United Kingdom: 4 Switzerland: 2 Netherlands: 1	Australia: 4 New Caledonia: 1	Bangladesh: 2 China: 2 Japan: 1 Bhutan: 1 Sri Lanka:1		
Lao PDR	n = 0	n = 33	n = 4	n = 2	n = 0	n = 2
		United Kingdom: 20 France: 13	Australia: 4	Japan: 2		Gabon: 1 Kenya: 1
Myanmar	n = 0	n = 7	n = 1	n = 4	n = 0	n = 0
		France: 4 United Kingdom: 3	Australia: 1	India: 3 Japan: 1		
Timor Leste	n = 0	n = 1	n = 1	n = 0	n = 0	n = 0
		United Kingdom: 1	Australia: 1			
Brunei	n = 0	n = 0	n = 0	n = 0	n = 0	n = 0

The total number of collaborations outside SEA has been shown to strongly correlate with traditional indices of research performance such as the number of publications (r = 0.916, p = 0.000), number of publications in journals with IF (r = 0.909, p = 0.000), and FWCI (r = 0.691, p = 0.019). It also has a strong correlation with citation (r = 0.774, p = 0.005), usage (r = 0.825, p = 0.002), captures (r = 0.870, p = 0.000), and social media (r = 0.927, p = 0.000) (Table 4).

**Table 4:** Correlation analysis of collaborations and research performance. * Strong correlation ** Very strong correlation IF: impact factor

Collaboration	Research performance	Spearman’s Rho Coefficient	p value
Total Collaboration	Number of publications	0.916	0.000**
	Number of publications in journals with IF	0.909	0.000**
	Field Weighted Index	0.691	0.019*
	Citation	0.774	0.005**
	Usage	0.825	0.002**
	Captures	0.870	0.000**
	Mentions	0.589	0.057
	Social media	0.927	0.000**

## Review

Discussion

Our study explored the research performance of SEA nations on viral infections of the nervous system. A total of 542 articles have been published in the major databases which have been searched in this study. Of these 542 articles, 466 or 86% have been published in journals with IF. As with other bibliometric studies on other neurological conditions in SEA, Singapore, Malaysia, and Thailand have the greatest contribution to the research output of the region [12-20]. More than 80% of publications from these countries were published in journals with IF. The Philippines was the third country in SEA with the greatest number of research on viral infections of the nervous system. Even though Singapore, Vietnam, Cambodia, and Laos have a smaller number of publications, more than 80% of articles coming from these countries have been published in journals with IF. The average FWCI of the articles from Singapore, Vietnam, Cambodia, as well as Malaysia and Thailand, were noted to be more than one. This indicated that the performance of these researches is comparable to their global benchmark.

The most commonly researched topic was Japanese encephalitis, which is the most important cause of viral encephalitis in the region. Research on epidemiology, diagnosis, and prevention as well as the economic impact of Japanese encephalitis have been done due to its endemicity in the region, the lack of available treatment, and its tendency to have major outbreaks every 2-15 years [27]. This was followed by rabies and other non-specific viral encephalitides.

Surprisingly, GDP per capita and the population did not correlate with research productivity in this region. However, the % GDP which was allotted for research and development was found to have a positive correlation to the number of researches produced as well as the impact of these researches in terms of citations. This finding was also noted in other bibliometric studies in the region indicating that as more resources are allocated for research, the quantity and quality of researches increase [12,14,15,20].

The majority of the studies were descriptive studies. This type of research requires less time and resources which may be the reason why it was the most common research design used. This was followed by laboratory studies that looked into viral genomes, sequencing, and evolution. In 2005, the creation of the South East Asia Infectious Disease Clinical Research Network which was a collaboration between Thailand, Vietnam, and Indonesia with support from the United States National Institutes of Allergy and Infectious Diseases and the Wellcome Trust of the United Kingdom helped with laboratory capacity building within the region not only by providing new equipment and reagents but also with staff training, quality control, and biosafety. This further facilitated collaboration and data sharing between the countries involved [28,29]. This may have aided in increasing the quantity and quality of laboratory research in SEA despite the disparity in resources between the countries in the region.

This study has shown a strong correlation between collaboration with countries outside SEA and the number of research produced and its performance in terms of citations, usage, captures, and social media. These collaborations were mostly with countries that had historical ties with the SEA nations at one point in time. The educational institutions as well as the research partnerships which were established and maintained during these occupations may have significantly contributed to the research performance of the region. It is noteworthy, however, that this study has also shown that collaboration with other neighboring Asian countries such as Japan, China, and South Korea also contributed to the research performance of the region. In the 2019 Global Research Report in South and SEA by the Institute for Scientific Information, the emerging networks between SEA countries and other countries in the Asia-Pacific region in terms of research collaboration were also noted. In the same report, the average category normalized citation impact of SEA nations was 1.35. However, when publications with international collaborations were removed, the average citation impact of the region decreased to 0.635 [30]. As was also seen in this study, research performance in SEA is highly dependent on international collaborations to increase the impact of the studies produced.

The number of neurologists in the country also showed a positive correlation with research productivity and impact. However, the population served per neurologist did not show any correlation. This was also seen in other studies on research productivity [12,15,18]. As with other medical specialties, the “Publish or Perish” mentality appears to be evident in the field of neurology. Publications are required for career advancements in terms of promotion as well as job retention, especially in academic and training institutions. Residents and fellows are also expected to participate in research during their training. Training institutions and neurologic societies, in turn, support research activities by providing training and expert consultation as well as enabling collaboration and funding opportunities.

To our knowledge, this was the first study to look into the performance of SEA countries with regard to research on viral infections of the nervous system. An extensive search and analysis were done in major databases and the articles were carefully selected and analyzed. However, the limitations of this study include the non-inclusion of studies with no available full-text document online, articles published in non-indexed journals, as well as those which were not written in English. Due to these exclusion criteria, we surmise that many studies were not counted and the true number of researches may have been underestimated.

Research capacity building is crucial in the control and management of these viral infections of the nervous system, particularly in SEA where these diseases have been noted to emerge and re-emerge. It is important that researches on these diseases especially with regard to their pathophysiology, epidemiology, prevention and treatment be done before they cause significant outbreaks and spread to other regions. Research endeavors should be encouraged and supported through better resource allocation. Collaborations with other countries both within and outside SEA should be strengthened since it can increase the scientific performance of the region in spite of the differences between laboratory capacity and other resources. Given the results of this study, future researchers can also look into the scientific performance of the region on other significant etiologies of nervous system infections such as parasites, fungi, and prions as these are also seen in the region.

## Conclusions

The findings of this study have shown that although the number of researches coming from SEA is relatively low in terms of number, the quality and impact of these researches may be comparable to other research output from western countries. The percentage of GDP allotted for research and development, the number of neurologists, and collaborations positively affect the research performance of the region. This should encourage researchers in SEA to produce more high-quality studies, not only on viral infections but also on the other infectious causes of neurologic diseases. Improving resource allocation as well as increasing collaboration and data sharing between SEA nations and other countries may support this endeavor.
